# 15. ON THE ROAD TO CURING SCHIZOPHRENIA

**DOI:** 10.1093/schbul/sby014.058

**Published:** 2018-04-01

**Authors:** Cynthia Shannon Weickert

**Affiliations:** Neuroscience Research Australia: Schizophrenia Research Laboratory

## Abstract

**Overall Abstract:** I began my journey to find out what caused schizophrenia around the time me and my twin brother, Scott David, turned 17. My first step was to conceptualize schizophrenia as a biological, cellular and molecular brain problem. This guided my choice of undergraduate and graduate study. I quickly realized that schizophrenia was not a “genetic” disease, nor was it an “environmental” disease, it was both. I prioritized studying RNA as was the active genome, the subcellular substrate where genes and environment interact. Guided from my own experience of watching my normal twin be tortured by schizophrenia in his teens, I sought to find answers by studying the mammalian brain as it developed and changed during adolescence. For my post-doctoral fellowship, I joined the laboratory of Joel Kleinman, who has the largest and best characterized human brain collection of people with schizophrenia in the world. Along my journey, while at NIMH in the USA, I discovered changes in neuronal growth factors and hormone receptors during stages of postnatal life and in the brains of people with schizophrenia compared to controls using the classical hypothesis-driven approach. Since I moved to Sydney Australia, I choose a different, more open-minded approach and let the brains of those who suffered “tell me what happened to them”, using a modern, sensitive discovery-driven RNAseq approach. When I listened, more carefully at the molecular level, what I found told me that I may be headed down the wrong path with my research and that I needed to change direction. It suggested that the emphasis I placed on development molecules maybe in some ways blinding me from more clearly seeing the neuropathology that existed only in only some people at the time of death. I found elevated inflammatory cytokine mRNAs in ~40% in the brains of those diagnosed with chronic schizophrenia. In this talk, I will review my latest discoveries on neuroinflammation in schizophrenia including evidence of gliosis, blood-brain barrier (BBB) changes and increased white blood cells in the brains of some with schizophrenia. Today, many of my fellow seekers including geneticists (Chr 6, MHC locus) and epidemiologists (maternal infection) and “animal modelers” (poly I:C) are suggesting that the cause of schizophrenia may involve changes to the immune system. These new discoveries suggest that very first steps I took may have been wrong, that perhaps I should have become an immunologist rather than a neurobiologist. However, from my current vantage point, I believe that even if a fault in the immune system plays a role in the causality of schizophrenia, that neurons are the major protagonist behind the manifestation of schizophrenia. The road ahead suggests that we, as a field, need to do some more trial blazing research that conceptualizes schizophrenia as not a “brain” disease and not as an “immune” disease, but as both.

